# Supplementary material for: Neural Signatures of Error Processing in Depressed Adolescents with Comorbid Non-Suicidal Self-Injury (NSSI)
Source: Biomedicines. 2022 Dec 8;10(12):3188. doi: 10.3390/biomedicines10123188 (PMC9775576; doi:10.3390/biomedicines10123188)
Supplement: Supplementary file 1 [file biomedicines-10-03188-s001.zip › biomedicines-2018933-supplementary.pdf]

Supplementary Material

Neural Signatures of Error Processing in Depressed Adolescents with Comorbid Non-Suicidal Self-Injury (NSSI)

Correlational analyses between neural activations and behavioural responses

To examine significant associations between neural activations and behavioural responses (number of commission errors and reaction times) during fMRI in the patients, differential fMRI parameter estimates of significant peak voxel activations from between-group comparisons were extracted for correlation analyses, namely from dACC and IFG as key structures of neural error processing.

We observed a significant negative correlation between the individual number of commission errors and individual neural activations of the contrast `incorrect minus correct NoGo trials` within the dACC in MD-only ($r = -0.653$; $p = {}^*0.021$). After correcting for one outlier in MD-NSSI with an extraordinary high number of commission errors ($n = 48$), a reversed, significant correlation was seen in MD-NSSI ($r = 0.574$; $p = {}^*0.040$). Corresponding graphs are shown in **Figure S1**. No significant correlations were detected regarding reaction times.

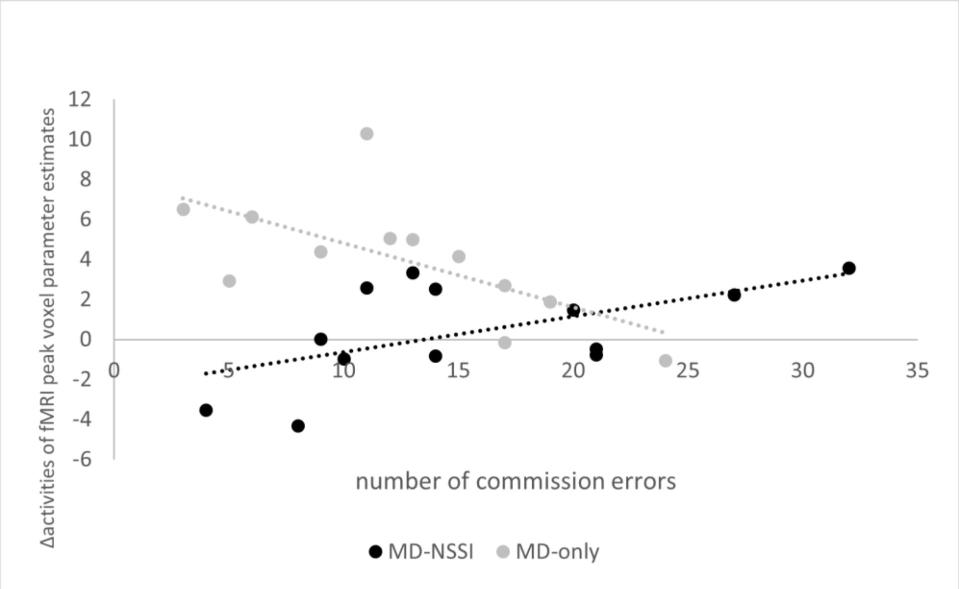

Figure S1: Scatterplot of numbers of commission errors in correlation to differential neural activities within midline dorsal anterior cingulate cortex (dACC, peak voxel MNI coordinates: x/y/z = 10/22/38)) presented with corresponding trend lines in depressed adolescents with

comorbid non-suicidal self-injury (MD-NSSI; n = 13) and in adolescents with major depression (MD-only; n = 13).

A relationship between differential fMRI parameter estimates and commission errors with corresponding reaction times in NoGo trials was also apparent in the left inferior frontal gyrus (IFG, pars opercularis). However, differential activities were significantly correlated only in MD-only with number of commission errors/reaction time (r = −0.700; p = *0.011/r = 0.645; p = *0.023), while opposite correlations were not significant in MD-NSSI.

Table S1: Significant neural activations (p ≤ 0.05; minimum cluster size of k = 183 contingously significant voxels at p < 0.005, uncorrected) of the contrast ′incorrect minus correct incongruent NoGo trials′ separately for healthy adolescent controls (HC; n = 14), adolescents with major depression (MD-only; n = 12) and depressed adolescents with comorbid non-suicidal self-injury (MD-NSSI; n = 14):

	BA	*Anatomic label*	*L/R/M*	*cluster size k (Vx)*	*Z*	*MNI*		
						x	*y*	*z*
HC	24	dACC	M	1688	7.58	4	22	36
	32	aMCC	M	#	6.87	0	42	24
	47	anterior insula	L	#	7.56	−38	20	0
	47	anterior insula	R	#	7.46	36	24	2
	48	IFG, pars opercularis	L	#	6.56	−50	14	2
	48	IFG, pars opercularis	R	#	7.01	48	16	0
	45	middle frontal gyrus	L	#	4.71	−40	36	20
	45	middle frontal gyrus	R	#	6.24	42	36	24
	8	SMA	M	#	6.61	4	18	50
	6	precentral gyrus	L	2744	3.25	−28	−20	62
	2	postcentral gyrus	L	#	5.13	−48	−30	48
	40	inferior parietal cortex	L	#	6.99	−44	−46	50
	40	supramarginal gyrus	L	#	5.93	−58	−48	32
	40	inferior parietal cortex	R	2525	6.31	50	−38	50
	48	supramarginal gyrus	R	#	6.59	60	−40	28
	40	angular gyrus	R	#	4.79	34	−54	38
	7	precuneus	L	588	3.74	−10	−64	40
	7	cuneus	R	#	4.70	16	−66	40
	22	middle temporal gyrus	L	191	3.56	−58	−42	2
	20	middle temporal gyrus	R	506	5.34	58	−24	−10
	0	midbrain	M	2212	5.96	−6	−26	−6
	0	caudate nucleus	L	#	4.07	−12	6	10
	0	caudate nucleus	R	#	4.55	12	0	16
	0	thalamus	L	#	3.83	−12	−18	8
	0	thalamus	R	#	5.25	8	−14	0

MD-only	24	dACC	M	#	6.73	0	22	38
	32	aMCC	M	16739	6.22	4	36	22
	47	anterior insula	L	#	7.74	−36	22	0
	38	anterior insula	R	#	5.72	44	14	−8
	48	IFG, pars opercularis	L	#	6.72	−48	10	4
	48	IFG, pars triangularis	L	#	6.12	−38	28	20
	32	SMA	M	#	6.90	−2	20	44
	48	IFG, pars triangularis	R	1067	3.71	44	24	32
	46	middle frontal gyrus	R	#	4.80	40	36	28
	3	postcentral gyrus	L	3545	4.00	−44	−20	54
	40	inferior parietal cortex	L	#	5.78	−58	−40	38
	48	supramarginal gyrus	L	#	5.96	−48	−42	36
	48	Rolandic operculum	L	#	4.70	−48	−24	24
	40	inferior parietal cortex	L	#	4.87	−36	−50	42
	40	inferior parietal cortex	R	772	4.21	36	−52	48
	7	angular gyrus	R	#	4.04	32	−56	46
	0	precuneus	L	488	4.40	−10	−60	46
	7	precuneus	R	#	3.58	6	−68	42
MD-NSSI	32	dACC	M	4570	6.71	−8	26	28
	32	aMCC	M	#	5.23	10	38	30
	6	SMA	M	#	6.87	−4	14	54
	46	superior frontal gyrus	R	#	3.70	22	48	24
	48	anterior insula	L	2542	7.08	−42	16	0
	46	IFG, pars triangularis	L	#	3.98	−44	48	6
	48	anterior insula	R	1894	6.05	36	18	4
	46	IFG, pars triangularis	R	270	4.14	40	50	−2
	40	inferior parietal cortex	L	274	4.40	−52	−38	48
	48	supramarginal gyrus	L	404	3.53	−58	−26	26
	48	Rolandic operculum	L	#	3.41	−44	−30	24
	0	midbrain	M	2193	5.40	−6	−24	−10
	0	thalamus	L	#	4.47	−8	−18	0
	0	thalamus	R	#	4.86	6	−20	2
	30	cerebellum/vermis	M	#	3.96	8	−40	−18

BA: Brodman area; L/R/M: left/right/midline; Z: z-score of standard norm distribution; k: number of voxels (Vx); MNI: Montreal Neurological Insitute (x-, y-, z-coordinates are provided in mm); dACC: dorsal anterior cingulate cortex; aMCC: anterior midcingulate cortex; #: maximum part of cluster above; ant. : anterior; IFG: inferior frontal gyrus; SMA: supplementary motor area.

Table S2: Significant neural activations (p ≤ 0.05; minimum cluster size of k = 183 continguously significant voxels at p < 0.005, uncorrected) of the contrast ′incorrect minus correct incongruent Go trials′ separately for healthy adolescent controls (HC; n = 14), adolescents with major depression (MD-only; n = 13) and depressed adolescents with comorbid non-suicidal self-injury (MD-NSSI; n = 14):

	BA	*anatomic label*	*L/R/M*	*cluster size k (Vx)*	*Z*	*MNI*		
						x	*y*	*z*
**HC**	9	dACC	R	8987	5.38	4	16	42
	32	dACC	L	#	4.75	−10	16	34
	47	anterior insula	R	#	4.42	40	22	4
	46	middle frontal gyrus	R	#	4.71	40	38	28
	6	SMA	M	#	6.04	6	0	62
	6	precentral gyrus	R	#	5.17	42	2	46
	48	IFG, pars opercularis	R	#	4.62	56	14	0
	47	IFG, pars orbicularis /anterior insula	L	722	3.72	−42	22	−8
	48	IFG, pars triangularis	L	297	3.76	−40	18	28
	6	precentral gyrus	L	392	3.72	−32	−4	60
	4	postcentral gyrus	L	#	3.25	−42	−26	58
	40	inferior parietal cortex	L	1316	4.76	−40	−48	54
	7	intraparietal sulcus	L	#	4.68	−32	−64	52
	40	supramarginal gyrus	L	#	3.82	−52	−48	30
	39	angular gyrus	L	#	3.62	−48	−50	30
	40	supramarginal gyrus	R	2406	5.25	64	−38	34
	40	inferior parietal cortex	R	#	3.96	36	−52	40
	7	angular gyrus	R	#	3.60	34	−68	46
	21	middle temporal gyrus	R	#	3.42	56	−30	−6
	7	precuneus	M	3972	3.98	4	−62	42
	17	occipital cortex	M	#	4.25	6	−76	−4
	0	thalamus	R	384	4.01	10	−12	0
	0	cerebellum/vermis	M	957	4.78	−6	−56	−28
**MD-only**	0	pCC	R	177	4.52	14	−24	40
	24	dACC	M	13377	6.50	−4	24	36
	24	aMCC	M	#	5.22	6	28	24
	45	IFG, pars triangularis/ anterior insula	L	#	6.54	−48	32	14
	48	IFG, pars opercularis	L	#	6.14	−48	14	6
	45	IFG, pars triangularis/ anterior insula	R	#	5.19	50	30	4
	48	IFG, pars opercularis	R	#	6.54	52	14	8
	4	precentral gyrus	L	#	5.69	−50	10	38

	6	precentral gyrus	R	#	4.59	34	2	48
	8	sup. frontal gyrus	R	#	5.43	22	10	52
	8	superior frontal gyrus	L	#	4.14	−22	12	50
	32	medial frontal gyrus	R	#	4.37	10	50	24
	32	SMA	M	#	5.29	4	16	48
	40	supramarginal gyrus	R	1766	4.06	54	−36	40
	22	angular gyrus	R	#	3.92	56	−56	28
	22	superior temporal gyrus	R	#	4.12	62	−44	14
	21	middle temporal gyrus	R	#	3.73	62	−46	6
	48	Rolandic operculum	R	#	3.31	50	−26	22
	48	supramarginal gyrus	L	1733	5.92	−50	−40	34
	39	angular gyrus	L	#	3.42	−54	−64	26
	21	middle temporal gyrus	L	#	3.33	−48	−56	22
	7	superior parietal cortex	L	#	3.21	−24	−58	50
	40	inferior parietal cortex	L	#	4.31	−36	−46	42
	0	precuneus	L	#	3.44	−12	−50	52
	0	precuneus	R	255	3.51	14	−48	50
	0	occipital cortex	L	374	4.02	−16	−64	30
	0	midbrain	M	297	3.85	−4	−26	−2
	0	thalamus	L	#	3.12	−6	−12	0
	0	thalamus	R	#	3.07	6	−18	0
**MD-NSSI**	24	dACC	M	33781	6.77	−2	20	38
	48	IFG, pars opercularis/ anterior insula	L	#	6.34	−46	14	2
	48	anterior insula	R	#	3.75	44	12	0
	46	middle frontal gyrus	L	#	5.56	−38	36	24
	46	middle frontal gyrus	R	#	4.57	38	36	26
	32	SMA	M	#	7.07	0	12	48
	6	precentral gyrus	L	#	5.84	−38	−6	50
	6	precentral gyrus	R	#	3.79	36	−8	58
	2	postcentral gyrus	R	#	4.46	44	−36	58
	48	supramarginal gyrus	R	#	5.82	58	−22	24
	48	supramarginal gyrus	L	#	5.21	−62	−40	28
	40	sulcus intraparietalis	R	#	4.94	34	−40	48
	40	inferior parietal cortex	L	#	4.39	−40	−40	43
	48	inferior parietal cortex	R	#	4.94	34	−40	48
	7	precuneus	M	#	5.91	−10	−64	48
	17	occipital cortex	M	#	5.49	4	−74	12
	0	thalamus	R	266	4.82	14	−20	8

BA: Brodman area; L/R/M: left/right/midline; Z: z-score of standard norm distribution; MNI: Montreal Neurological Insitute (x-, y-, z-coordinates are provided in mm); k: number of voxels (Vx); dACC: dorsal anterior cingulate cortex; #: maximum part of cluster above; SMA: supplementary motor area; IFG: inferior frontal gyrus; pCC: posterior cingulate cortex; aMCC: anterior midcingulate cortex.
